# Assessment of cardiac output changes using a modified FloTrac/Vigileo™ algorithm in cardiac surgery patients

**DOI:** 10.1186/cc7739

**Published:** 2009-03-04

**Authors:** Alban Senn, Danny Button, Andreas Zollinger, Christoph K Hofer

**Affiliations:** 1 Institute of Anaesthesiology and Intensive Care Medicine, Triemli City Hospital Zurich, Birmensdorferstrasse 497, 8063 Zurich, Switzerland

## Abstract

**Introduction:**

The FloTrac/Vigileo™ (Edwards Lifesciences, Irvine, CA, USA) allows pulse pressure-derived cardiac output measurement without external calibration. Software modifications were performed in order to eliminate initially observed deficits. The aim of this study was to assess changes in cardiac output determined by the FloTrac/Vigileo™ system (FCO) with an initially released (FCOA) and a modified (FCOB) software version, as well as changes in cardiac output from the PiCCOplus™ system (PCO; Pulsion Medical Systems, Munich, Germany). Both devices were compared with cardiac output measured by intermittent thermodilution (ICO).

**Methods:**

Cardiac output measurements were performed in patients after elective cardiac surgery. Two sets of data (A and B) were obtained using FCOA and FCOB in 50 patients. After calibration of the PiCCOplus™ system, triplicate FCO and PCO values were recorded and ICO was determined in the supine position and cardiac output changes due to body positioning were recorded 15 minutes later (30° head-up, 30° head-down, supine). Student's t test, analysis of variance and Bland-Altman analysis were calculated.

**Results:**

Significant changes of FCO, PCO and ICO induced by body positioning were observed in both data sets. For set A, ΔFCOA was significantly larger than ΔICO induced by positioning the head down. For set B, there were no significant differences between ΔFCOB and ΔICO. For set A, increased limits of agreement were found for FCOA-ICO when compared with PCO-ICO. For set B, mean bias and limits of agreement were comparable for FCOB-ICO and PCO-ICO.

**Conclusions:**

The modification of the FloTrac/Vigileo™ system resulted in an improved performance in order to reliably assess cardiac output and track the related changes in patients after cardiac surgery.

## Introduction

Cardiac output is monitored in critically ill patients to assess cardiac function in order to maintain adequate tissue perfusion. In order to accomplish this task the thermodilution technique using a pulmonary artery catheter has been used for decades as the clinical standard. However, based on results of different studies, its use has been questioned and there is an ongoing debate on its impact on patient outcome [1-3]. Several alternative, less invasive techniques are available today, with cardiac output derived from pulse pressure being one of the most used methods [4].

Continuous pulse contour analysis by the PiCCOplus™ system (Pulsion Medical Systems, Munich, Germany) assesses arterial pressure waveforms using a specific thermistor tipped catheter typically inserted into the femoral artery. Cardiac output is calculated using an algorithm measuring the area under the curve of the systolic pressure wave after calibration by transpulmonary thermodilution. Moreover, the calibration process is used to adapt for individual vascular compliance. The PiCCOplus™ system has been repeatedly shown to reliably assess for cardiac output in different clinical situations [5-7]. However, inaccurate measurements during haemodynamic changes were observed with the initial pulse contour algorithm [8] and the technique has been modified to better address the aortic compliance in these situations [9,10].

The recently introduced FloTrac/Vigileo™ system (Edwards Lifesciences, Irvine, CA, USA) on the other hand allows the cardiac output to be determined continuously using pulse wave analysis without external calibration. It samples pressure wave signals using a standard peripheral arterial line. The standard deviation of pulse pressure is empirically correlated to the stroke volume based on patient characteristics after automatic adjustment for actual vascular compliance and displayed as continuous cardiac output. Initial evaluation studies on the FloTrac/Vigileo™ system revealed conflicting results [11-13].

The observed weak or only fair agreement between the new system and a reference technique may be partly explained by the fact that adaption for changes of vascular compliance at 10 minute intervals may miss haemodynamic changes during that time window. Consequently, the FloTrac/Vigileo™ system with its underlying algorithm has been improved and – as a major modification – the time window was reduced to one minute (Software version 1.07 and higher). Consecutive studies using a modified FloTrac/Vigileo™ system showed improved results [14-18]. However, methodological issues regarding the study design (i.e. set-up of monitoring devices, predefined measurement points during the perioperative course of cardiac surgery, measurements taken early after cardiopulmonary bypass) may impede a reliable trend analysis. Moreover, comparisons of different FloTrac/Vigileo™ software versions in a clearly defined setting during haemodynamic changes have not been performed so far.

The aim of the present study was to assess cardiac output and the related changes determined by two pulse contour analysis devices after induction of haemodynamic changes by body positioning. The FloTrac/Vigileo™ system with an initially released and a modified software version, as well as the PiCCOplus™ system were used in patients after off-pump coronary artery bypass grafting. Both devices were compared with intermittent thermodilution.

## Materials and methods

### Patients and setting

With local ethics committee approval, patients scheduled for elective off-pump coronary artery bypass grafting were enrolled in this study after written informed consent was obtained. Exclusion criteria were reduced left and right ventricular function (ejection fraction less than 40%), preoperative dysrhythmias, severe valvular heart diseases, intracardiac shunts, pulmonary artery hypertension, severe arterial occlusion disease and body weight less than 40 kg. A total of 50 patients were enrolled and cardiac output was determined by the FloTrac/Vigileo™ system (FCO), 25 patients using FloTrac/Vigileo™ software version 1.03 (FCOA) and 25 patients using software version 1.07 (FCOB), set A and B, respectively. The sample size was determined on the hypothesis of an expected standard deviation of 8% for cardiac output values and an expected difference in the range of the standard deviation between the values of the different measurement techniques (α = 0.05 and power > 0.9).

### Routine perioperative management

Perioperative management was performed according to institutional standards. Routine monitoring (Philips IntelliVue™ Monitoring; Philips Medical Systems, Andover, MA, USA) during the entire perioperative period included pulse oxymetry, five-lead ECG, invasive blood pressure measurement via a peripheral radial arterial and central venous pressure (CVP) assessed by standard transducers (Truewave™ PX; Edwards Lifesciences, Irvine, CA, USA). At the time the study was performed the PiCCOplus™ system was the routine continuous cardiac output monitoring system in the selected patient group according to institutional standards. After induction of anaesthesia a 4F thermistor-tipped arterial catheter (Pulsiocath™ thermodilution catheter) was inserted into the left femoral artery, its tip advanced to the abdominal aorta and connected to the stand-alone computer PiCCOplus™ (Version 6.0; Pulsion Medical Systems, Munich, Germany). Intraoperative continuous cardiac output measurement was initiated after the initial calibration of the system using a 20 ml ice-cold normal saline injection via a standard central venous catheter in triplicate.

### Study setting

Measurements were started in the postoperative period after transfer of the patients to the intensive care unit. The patients remained sedated during the study period using propofol and remifentanil infusion. Rocuronium (0.2 to 0.5 mg/kg/hour) for neuromuscular blockade was administered when needed. Mean arterial pressure (MAP) was kept between 65 and 75 mmHg adjusting the patient's noradrenaline dose (0 to 10 μg/minute). No other catecholamines had to be used during the study period. In order to maintain regular heart rhythm in all patients, fixed external pacing at a heart rate (HR) between 80 and 90 beats/minute was used. The patients' lungs were mechanically ventilated using a volume-controlled mode (tidal volume = 6 to 8 ml/kg, respiratory frequency = 12 breaths/minute, positive end-expiratory pressure = 5 cmH_2_O, peak inspiratory pressure = 18 to 25 cmH_2_O) in order to achieve normoventilation (partial pressure of carbon dioxide = 4 to 4.5 kPa).

Weaning from the ventilator was started after completion of the study protocol. The femoral PiCCO™ catheter introduced after induction of anaesthesia was connected to the stand-alone monitor PiCCOplus™ (computer version 5.2.2; Pulsion Medical Systems, Munich, Germany) and calibrated according to the manufacturer's instruction. A FloTrac™ sensor kit was connected to the radial arterial line and connected to the Vigileo™ (Edwards Lifesciences, Irvine, CA, USA). For set A, software version 1.03 was used and for set B software version 1.07 was used. Individual patient data were entered including age (years), gender, body weight (kg) and height (cm). Measurements were initiated after checking the arterial line waveform fidelity and zeroing the system at mid-axillary level to ambient pressure.

### Pulse wave analysis algorithms

Cardiac output is assessed by the FloTrac/Vigileo™ and PiCCOplus™ systems using different proprietary algorithms. These have been described in detail elsewhere [6,15]. Briefly, the calculation of cardiac output by the FloTrac/Vigileo™ system is based on the contribution of pulse pressure on cardiac output being proportional to the standard deviation of arterial pulse pressure. The influence of vascular resistance and compliance on cardiac output is considered manually based on entered patient data. Thus, there is no need for external calibration. By contrast, the PiCCOplus™ method relies on the work of Wesseling and colleagues [19] calculation of cardiac output by measuring the area under the curve of the systolic arterial pressure wave form and dividing this area by the aortic impedance after calibration by transpulmonary thermodilution. For adequate determination of cardiac output and the adjustment of individual aortic compliance, however, calibration by transpulmonary thermodilution is required.

### Study protocol

**Figure 1:**
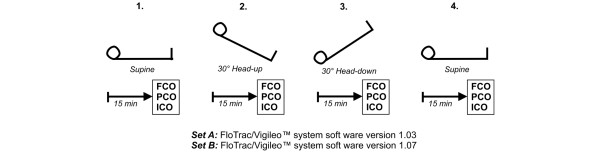
Study protocol. Set A was FCO assessed using the software version 1.03, and set B was FCO assessed using the software version 1.07. FCO = cardiac output assessed by the FloTrac/Vigileo™ device; ICO = cardiac output determined by the intermittent thermodilution; PCO = cardiac output assessed by the PiCCOplus™ system.

Haemodynamic measurements were performed in the following positions: in supine position (haemodynamic stability); 15 minutes after initiation of a 30° head-up position; 15 minutes after tilting the patient's bed to a 30° head-down position; and 15 minutes after adjusting the patient into a supine position again. At all four time points the following haemodynamic variables were recorded: MAP, HR, CVP, FCO and cardiac output readings from the PiCCOplus™ system (PCO). Immediately thereafter, triplicate transpulmonary thermodilution measurements of 20 ml normal iced saline solution were performed to determine cardiac output measured by intermittent thermodilution (ICO). Post-hoc systemic vascular resistance (SVR) was calculated (Figure 1). No recalibration of the PiCCOplus™ was performed during the study period.

### Statistics

All haemodynamic variables were recorded as a mean of three repeated measurements. Statistical analysis was performed using Statview 5.01^® ^Software (SAS Institute Inc., Cary, NC, USA) and SPSS^® ^10.0 (SPSS Inc., Chicago, IL, UK). Cardiac output changes were calculated as percentage deviation of the previous measurements. Analysis of variance for repeated measurements (*post-hoc* Bonferroni correction) was used to assess differences of haemodynamic variables during the study period. Paired student's t-test and Bland-Altman analysis (including percentage error according to Critchley and Critchley [20]) was performed to compare cardiac output values obtained by the different devices and cardiac output assessed by intermittent thermodilution. Unless otherwise stated, data are presented as mean ± standard deviation.

## Results

A total of 50 American Society of Anesthesiologists (ASA) physical status III patients with preserved left ventricular function undergoing elective cardiac bypass surgery were enrolled (Table 1). In total, 100 matched sets of data were obtained for both sets. Sociodemographic characteristics are summarised in Table 1.

**Table 1:** Sociodemographic data

	Set A	Set B	P value
Age (years)	64.7 (10.6)	66.6 (7.6)	0.386
Female/male ratio	5/20	6/19	0.791
BMI (kg/m^2^)	26.6 (3.5)	27.9 (3.6)	0.208
EURO score	4.0 (3.0)	4.0 (2.0)	0.635
Preoperative EF (%)	58.0 (11.6)	57.2 (9.2)	0.685
CABG off	4.0 (1.0)	4.0 (1.0)	0.430

BMI = body mass index; CABG off = off-pump coronary artery bypass grafting; EF = ejection fraction; EURO = European System for Cardiac Operative Risk Evaluation.

**Figure 2:**
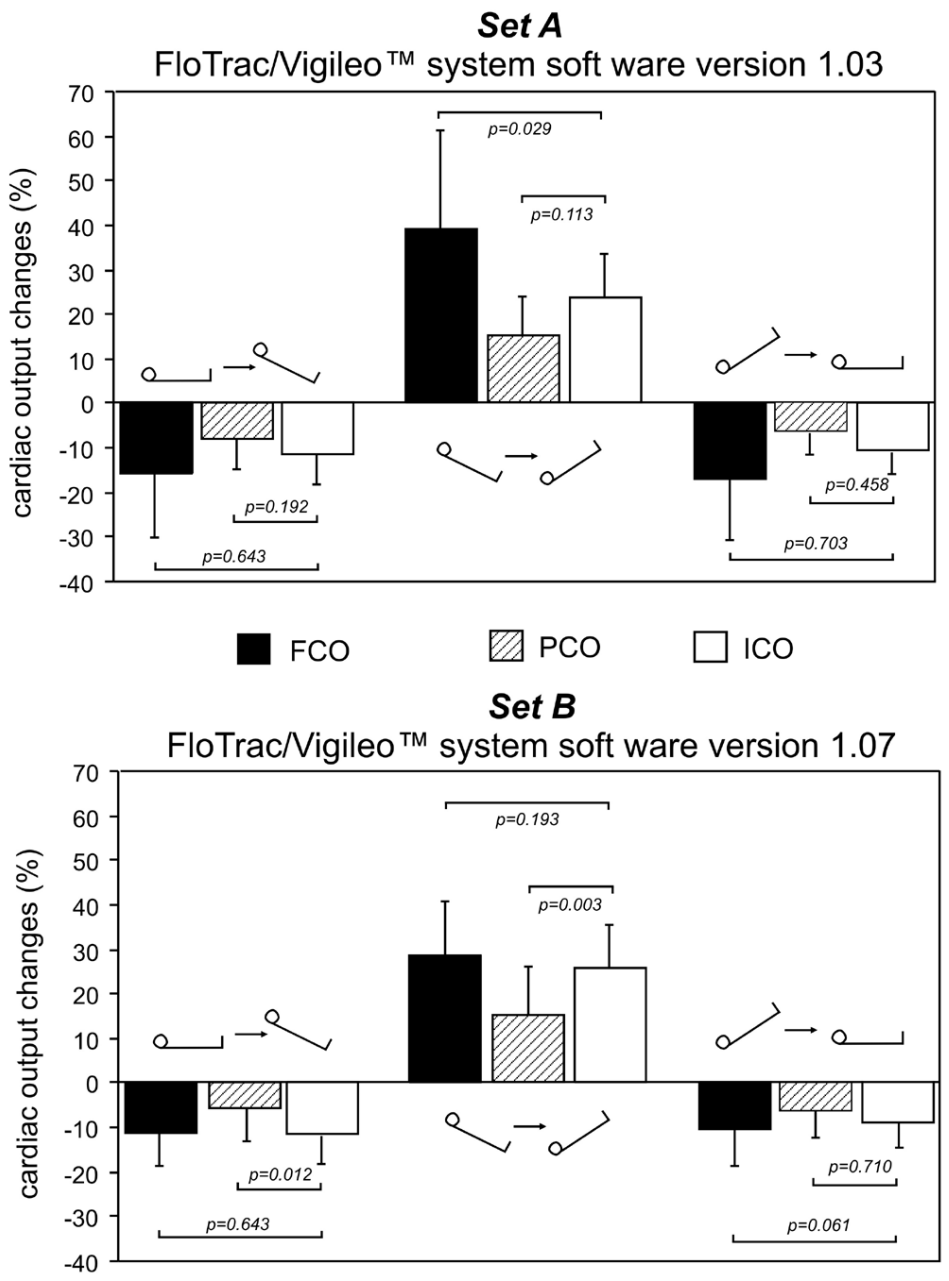
Cardiac output changes assessed during the study period. Set A was FCO assessed using the software version 1.03, and set B was FCO assessed using the software version 1.07. FCO = cardiac output assessed by the FloTrac/Vigileo™ device; ICO = cardiac output determined by the intermittent thermodilution; PCO = cardiac output assessed by the PiCCOplus™ system.

During the study period (Figure 1) significant haemodynamic changes after changing body positioning were observed for both sets of data (Table 2). Based on a fixed external pacing rate no changes in HR were observed. FCO, PCO and ICO significantly decreased after inducing a head-up position and significantly increased when tilting the patient into the head-down position. For all measurements, the direction of FCO, PCO and ICO changes were similar. For set A, there was a significant difference between the changes of FCO and ICO when inducing the 30° head-down position. For set B no significant differences were observed between FCO and ICO, whereas changes of PCO significantly underestimated ICO creating decreases and increases of cardiac output by body positioning (Figure 2).

**Table 2:** Haemodynamic variables during the study period

Set	Variables	Supine 1	Head-up	Head-down	Supine 2
A	FCO (L/minute)	5.5 (0.8)	4.7 (0.7)^a^	6.4 (1.1)^a, b^	5.5 (0.8) ^b, c^
	PCO (L/minute)	5.4 (0.8)	4.9 (0.8)^a^	5.8 (0.9)^a, b^	5.4 (0.9) ^b, c^
	ICO (L/minute)	5.7 (1.1)	5.0 (0.9)^a^	6.1 (1.1)^a, b^	5.6 (1.1) ^b, c^
	HR (beats/minute)	86 (8)	87 (10)	88 (9)	89 (9)
	MAP (mmHg)	75 (9)	68 (10)^a^	77 (10) ^b^	74 (10)
	CVP (mmHg)	10 (4)	6 (3)^a^	11 (4) ^a, b^	8 (3)^c^
	SVR (dyn sec/cm^-5^)	933 (170)	985 (207)	884 (235) ^b^	953 (145)

B	FCO (L/minute)	5.6 (1.0)	4.7 (0.9)^a^	5.1 (0.9)^a, b^	5.4 (1.2) ^b, c^
	PCO (L/minute)	5.5 (1.2)	4.7 (1.2)^a^	5.3 (1.4)^a, b^	5.5 (1.4) ^b, c^
	ICO (L/minute)	5.8 (1.1)	4.3 (1.1)^a^	5.0 (1.3)^a, b^	5.3 (1.5) ^b, c^
	HR (beats/minute)	88 (7)	67 (16)	82 (12)	89 (7)
	MAP (mmHg)	76 (5)	74 (11)	72 (7) ^b^	72 (8)
	CVP (mmHg)	9 (3)	7 (4)^a^	10 (3)^a, b^	8 (4)
	SVR (dyn sec/cm^-5^)	969 (175)	1001 (186)	960 (196)	1008 (195)

CVP = central venous pressure; FCO = cardiac output assessed by the FloTrac/Vigileo™ device; HR = heart rate; ICO = cardiac output determined by the intermittent thermodilution; MAP = mean arterial pressure; PCO = cardiac output assessed by the PiCCOplus™ system; SVR = systemic vascular resistance. Data are presented as mean (standard deviation).

^a ^*P* < 0.05 compared with supine 1 position; ^b ^*P* < 0.05 compared with head-up position; ^c ^*P* < 0.05 compared with head-down position.

**Figure 3:**
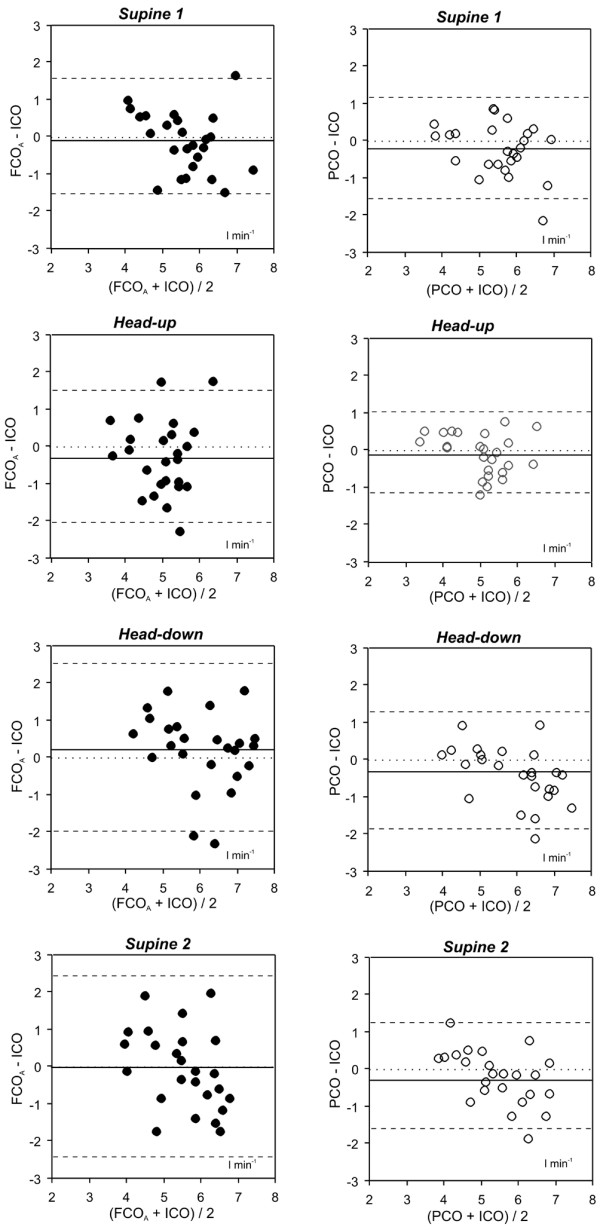
Bland-Altman analysis for set A: comparison of the FloTrac/Vigileo™ system and intermittent thermodilution. Solid line = mean bias; dashed lines = limits of agreement. FCOA = cardiac output assessed by the FloTrac/Vigileo™ system using the software version 1.03; ICO = cardiac output determined by the intermittent thermodilution; PCO = cardiac output assessed by the PiCCOplus™ system.

Bland-Altman analysis for the comparison of FCO and ICO revealed an overall mean bias and limits of agreement of -0.1 ± 2.1 L/minute for set A and -0.3 ± 1.1 L/minute for set B. Total percentage error for set A was 37.5%, analysis for each measurement point revealed a percentage error less than 30% for the initial assessment in a supine position (Table 3). A total percentage error of 21.6 % was found for set B. For all measurement points a percentage error less than 30% was found (Table 3) and limits of agreement were lower for set B when compared with set A (Figure 3).

**Table 3:** Bland-Altman analysis for FCO compared with ICO and PCO compared with ICO

	FCO-ICO				PCO-ICO			
	Set A		Set B		Set A		Set B	

	L/minute	%Error	L/minute	%Error	L/minute	%Error	L/minute	%Error

Supine 1	-0.1 (1.6)	28.7	-0.3 (1.2)	20.7	-0.2 (1.4)	24.6	-0.3 (1.2)	20.7
Head-up	-0.3 (1.8)	36.0	-0.3 (1.1)	25.6	-0.1 (1.1)	22.0	0.1 (1.0)	23.3
Head-down	0.2 (2.2)	36.1	-0.2 (1.1)^a^	22.0	-0.3 (1.6)	26.2	-0.4 (1.4)	28.0
Supine 2	0.0 (2.4)	42.8	-0.4 (1.0)^a^	18.9	-0.2 (1.4)	25.0	-0.3 (1.3)	24.5
Total	-0.1 (2.1)	37.5	-0.3 (1.1)^a^	21.6	-0.2 (1.4)	25.0	-0.2 (1.3)	25.5

FCO = cardiac output assessed by the FloTrac/Vigileo™ device; ICO = cardiac output determined by the intermittent thermodilution; PCO = cardiac output assessed by the PiCCOplus™ system. Data are presented as mean bias (limits of agreement). Percentage error (%Error) was calculated according to Critchley and Critchley [20]. ^a ^*P* < 0.05 compared with set A.

**Figure 4:**
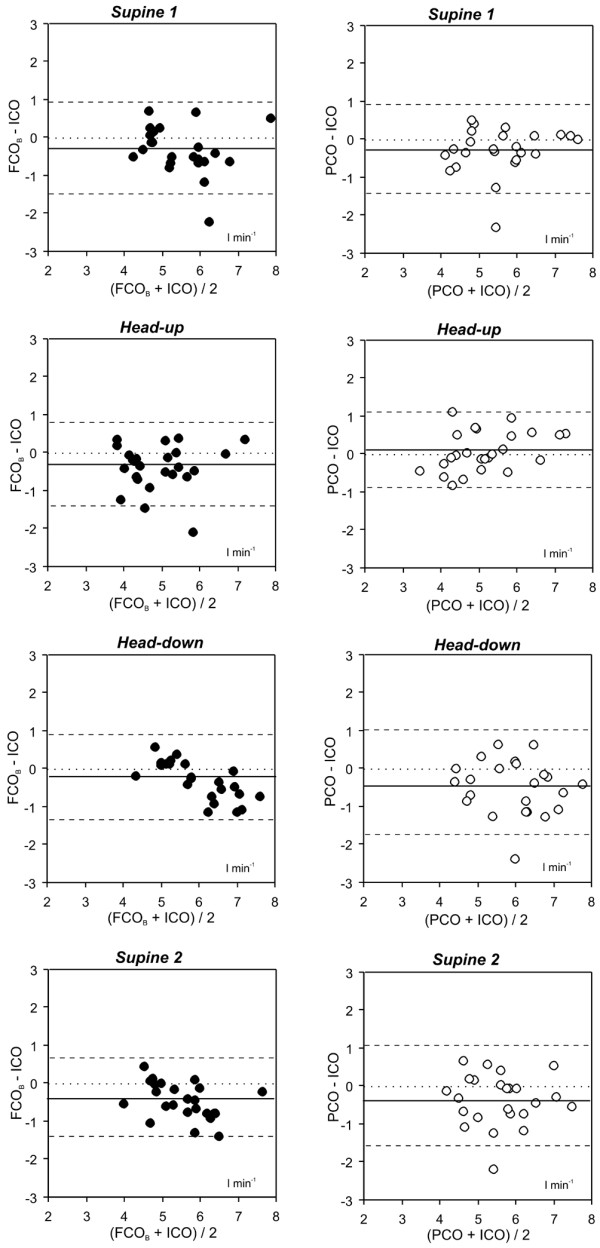
Bland-Altman analysis for set B: comparison of the PiCCOplus™ system and intermittent thermodilution. Solid line = mean bias; dashed lines = limits of agreement. FCOB = cardiac output assessed by the FloTrac/Vigileo™ system using the software version 1.07; ICO = cardiac output determined by the intermittent thermodilution; PCO = cardiac output assessed by the PiCCOplus™ system.

Comparison of PCO with ICO showed low limits of agreement and percentage error less than 30% for all measurement. Overall mean bias, limits of agreement and percentage error were -0.2 ± 1.4 L/minute and 25% for set A, and -0.2 ± 1.3 L/minute and 25.5% for set B, respectively (Table 3 and Figure 4).

## Discussion

In this study we evaluated cardiac output assessed by an initially released and a modified version of the FloTrac/Vigileo™, the PiCCOplus™ system and the bolus thermodilution – as reference technique – during haemodynamic changes induced by body positioning in patients after elective off-pump coronary artery bypass surgery. The modified FloTrac/Vigileo™ system showed an improved performance as compared with the early version of the system and measurements were as reliable as those performed by the PiCCOplus™ in this setting.

Cardiac output measurements by both less invasive monitoring devices used in this study rely on pulse pressure analysis. Although pulse pressure results from an interaction of the heart (cardiac output) and the vascular system, the influence of vascular properties and changes (resistance, impedance and compliance) have to be considered in the algorithms of the devices in order to reliably derive cardiac output. The FloTrac/Vigileo™ system calculates cardiac output analysing the impact of vascular tone on pressure and adjusts for actual vascular tone based on wave form analysis and patient characteristics. Therefore, compared with other pulse contour devices, it does not require any external calibration method or subsequent correction and may therefore avoid operator bias.

Studies validating the FloTrac/Vigileo™ system in cardiac surgery patients showed conflicting results using early software versions. In only one study were acceptable results regarding cardiac output measurements reported [11]. By contrast, Mayer and colleagues found in their study only a moderate agreement [12], whereas Sander and colleagues observed a weak agreement between the FloTrac/Vigileo™ system and intermittent thermodilution measurement in cardiac surgery patients [13]. Based on the initial clinical experience and the early validation studies the FloTrac algorithm was adapted to better address the issue of rapid haemodynamic changes.

The initial software version adjusted every 10 minutes for changes of vascular compliance, resistance and impedance. This time window was reduced to one minute for the software version 1.07 and higher as a major modification in order to improve cardiac output measurement. The studies performed with the modified FloTrac/Vigileo™ system revealed better results comparing cardiac output determined by this pulse wave analysis device with the standard intermittent thermodilution technique [14-18]. Our study group showed that perioperative performance of the FloTrac/Vigileo™ system, the PiCCOplus™ system and also the continuous cardiac output monitoring by the Vigilance monitoring based on the pulmonary artery catheter (Edwards Lifesciences, Irvine, CA, USA) was comparable in 31 patients undergoing elective coronary artery bypass surgery [15]. These findings were supported by a recently published study by Mayer and colleagues performed in a perioperative setting in 40 cardiac surgery patients [17]. Based on the study design, however, these studies were not able to directly demonstrate that the modifications of the FloTrac™ algorithm allow an improved monitoring and tracking of cardiac output changes. In the present study we induced haemodynamic changes by body positioning and using this approach we could show that cardiac output assessment by the initial FloTrac™ algorithm was insufficient in terms of increased limits of agreement and a percentage error higher than 30% (according to Critchley and Critchley [20]) when compared with the intermittent thermodilution method. By contrast, limits of agreement and percentage error were clearly reduced when cardiac output was measured by the modified algorithm. Moreover, changes of cardiac output were comparable with the reference technique.

Overall bias and limits of agreement of the PiCCOplus™ system were comparable with findings in previously published work [8-11]. For all data sets percentage error was lower than 30% when compared with intermittent thermodilution. Thus, our results demonstrate an adequate performance of the PiCCOplus™ system when inducing cardiac output changes by body positioning. In contrast to the FloTrac/Vigileo™ system, however, the PiCCOplus™ system showed a trend to underestimate cardiac output changes assessed by the reference technique. This was statistically significant for two changes in set B, but may not necessarily be considered as clinically relevant regarding the general performance of the PiCCOplus™ during the study. An operator error can be excluded, because the PiCCOplus™ system was recalibrated by the same observer according to the manufacturer's instructions. Moreover, optimal arterial signal quality was verified before each measurement sequence. However, it has been suggested, that for correct use the PiCCOplus™ system needs to be recalibrated at intervals of four to six hours when changes of vascular resistance are likely to occur or after major haemodynamic changes in order to maintain accuracy of cardiac output readings [4].

For the interpretation of the data presented in this study the following limitations have to be considered. We assessed cardiac output in a low-risk cardiac surgical group only. In clinical practice advanced monitoring techniques are typically used in patients at risk for major haemodynamic compromise.

In our study we induced cardiac output changes by body positioning. This approach does not necessarily reflect a clinically relevant haemodynamically unstable situation. However, head-down tilting manoeuvres have been increasingly propagated in the past few years in order to assess fluid responsiveness [21]. Moreover, this approach allowed us to perform a more reliable trend analysis compared with other comparative studies on cardiac output measurement.

Based on technical restrictions we were not able to simultaneously assess cardiac output using both FloTrac™ algorithms in the same patient group. However, the pre-defined study protocol was strictly followed in both patient groups and cardiac output measurements by the reference technique were comparable in both data sets.

We did not assess cardiac output in hyperdynamic situations [22], that is, patients with reduced peripheral resistance. Therefore, our findings may not be applicable to other situations and patients other than the population studied.

## Conclusions

In conclusion, our study results indicate that the modification of the FloTrac/Vigileo™ software with a reduced time window for vascular compliance adjustment resulted in an improved performance in order to assess cardiac output measurements and track the related alterations during haemodynamic changes induced by body positioning in patients after elective off-pump coronary artery bypass surgery.

## Key messages

• Software modification of the FloTrac/Vigileo™ system with a reduction of the time window for vascular adjustment from ten to one minute resulted in an improved performance of cardiac output measurement during haemodynamic changes in patients after cardiac surgery.

• The un-calibrated FloTrac/Vigileo™ system in its modified form allowed a reliable trend analysis when compared with intermittent thermodilution.

• Cardiac output can now be reliably assessed with a percentage error less than 30% using the FloTrac/Vigileo™ system with an upgraded software version as compared with intermittent thermodilution. The PiCCOplus™ system showed a comparable performance.

## Abbreviations

CVP: central venous pressure; FCO: cardiac output determined by the FloTrac/Vigileo™ system; HR: heart rate; ICO: cardiac output determined by determined by intermittent thermodilution; MAP: mean arterial pressure; PCO: cardiac output determined by the PiCCOplus™ system.

## Competing interests

This study was supported in part (set A) by a research grant from Edwards Lifesciences, Irvine, CA, USA. In the past, the Institute of Anaesthesiology and Intensive Care Medicine, Triemli City Hospital have held research grants from Pulsion Medical Systems, Munich, Germany, and Edwards Lifesciences, Irvine, CA, USA. CKH has previously received lecture fees from both companies, Pulsion Medical Systems, Munich, Germany, and Edwards Lifesciences, Irvine, CA, USA.

## Authors' contributions

CKH conceived of the study design and protocol, and participated in patient recruitment, measurements, data collection and statistical analysis, and drafted the manuscript. AS participated in patient recruitment, measurements and data collection, and drafted the manuscript. DB participated in patient recruitment, measurements and data collection. AZ conceived of the study design and protocol. All authors read and approved the final manuscript.
